# Assessment of changes in quality of life among patients in the SAVE Study - Sirolimus as therapeutic Approach to uVEitis: a randomized study to assess the safety and bioactivity of intravitreal and subconjunctival injections of sirolimus in patients with non-infectious uveitis

**DOI:** 10.1186/s12348-015-0044-1

**Published:** 2015-04-18

**Authors:** Erin M Vigil, Yasir Jamal Sepah, Anthony L Watters, Mohammad A Sadiq, Mehreen Ansari, Millena G Bittencourt, Mohamed A Ibrahim, Diana V Do, Quan Dong Nguyen

**Affiliations:** College of Arts and Sciences, Johns Hopkins University, 3400 North Charles Street, Baltimore, MD 21218 USA; Wilmer Eye Institute, Johns Hopkins University, 600 North Wolfe Street, Baltimore, MD 21287 USA; Ocular Imaging Research and Reading Center, Stanley M. Truhlsen Eye Institute, University of Nebraska Medical Center, 3902 Leavenworth Street, Omaha, NE 68105 USA; Emmes Corporation, 401 North Washington Street, Rockville, MD 20850 USA

**Keywords:** Sirolimus, Uveitis, Intravitreal, mTOR

## Abstract

**Background:**

The National Eye Institute 39-Question Visual Function Questionnaire (NEI VFQ-39) is an indicator of vision-related quality of life (QoL). The NEI VFQ-39 is used to assess the QoL in patients with non-infectious posterior uveitis, intermediate uveitis, or panuveitis, treated with subconjunctival (SCJ) or intravitreal (IVT) sirolimus as an immunomodulatory therapeutic (IMT) agent, delivered subconjunctivally (SCJ) or intravitreally (IVT) (the SAVE Study). Thirty subjects with non-infectious uveitis were randomized (SCJ:IVT, 1:1) for a prospective clinical trial. The 39-Question Visual Function Questionnaire (VFQ-39) was administered at baseline (BL), month 6 (M6), and month 12 (M12) visits. The survey measures self-reported vision health status for patients with chronic eye disease and assesses the effects of visual impairment on both task-oriented visual function and general health domains. In accordance to the NEI-VFQ Manual, each patient’s questionnaire was converted to a scaled score between 0 (worst) and 100 (best), and median scores were calculated for each of the subcategories and overall composite score at BL, M6, and M12. Wilcoxon signed-rank test was performed.

**Results:**

Twenty-six patients completed the VFQ-39 at BL and M6, whereas 23 patients completed it at M12. Patients showed a significant improvement in pooled composite scores from BL to M6 and BL to M12. Analysis by treatment groups showed that intravitreal injection of sirolimus is better tolerated.

**Conclusions:**

Sirolimus has demonstrated bioactivity as an IMT and corticosteroid-sparing agent to treat non-infectious uveitis. Patients receiving intravitreal injection of sirolimus showed overall improvement of vision-related health while those receiving subconjunctival injections did not. Larger randomized control trials with sirolimus are indicated to validate these results.

**Trial registration:**

ClinicalTrials.gov: NCT00908466

**Electronic supplementary material:**

The online version of this article (doi:10.1186/s12348-015-0044-1) contains supplementary material, which is available to authorized users.

## Background

Uveitis is an ocular disease that results from inflammation and tissue damage, which compromises the uvea of the eye [1]. Uveitis is the fourth most common cause of blindness among the working-age population in the developed world [2]. It is responsible for approximately 10% of the cases of blindness in USA [2]. Non-infectious uveitis may be an ocular manifestation of one of various autoimmune diseases, such as reactive arthritis, ankylosing spondylitis, Behçets syndrome, and inflammatory bowel disease, or it can limit exclusively to the ocular structures.

Topical, periocular, intraocular, and systemic corticosteroids are the mainstay of primary immunosuppressive therapy as well as the only United States Federal Drug Agency (US-FDA)-approved drug class in the United States for treatment of non-infectious uveitis [3]. However, corticosteroid treatment induces a high rate of adverse effects such as increased intraocular pressure, cataracts, Cushingoid syndrome, diabetes, osteoporotic bones, congestive heart failure, and metabolic disturbances [2,4]. Consequently, newer steroid-sparing agents (such as sirolimus, adalimumab, and gevokizumab) are being developed and evaluated for the treatment of non-infectious uveitis.

Sirolimus is an immunosuppressant that works through its inhibition of the mammalian target of rapamycin (mTOR) and subsequent inhibition of inflammatory cytokine production [5]. Sirolimus inhibits the inflammatory process and can be delivered both intravitreally and subconjunctivally. Subconjunctival and intravitreal sirolimus have demonstrated evidences of safety and efficacy in patients with non-infectious uveitis [6].

The National Eye Institute Visual Function Questionnaire 39-Item (NEI VFQ-39 or NEI VFQ-25 + additional items) is a self-administered survey that has been widely used to assess patient vision-related functioning. The NEI VFQ-39 survey contains 39 questions that evaluate 12 subscales of quality of life (QoL) including general health, general vision, ocular pain, near vision, distance vision, social function, mental health, role difficulty, dependency, driving, color vision, and peripheral vision. Each question has multiple choices that are scored on a five-, six-, or ten-point scale.

The NEI-VFQ has been used previously in several studies to assess the impact of ocular disorders and their treatments on visual function. These studies indicate that the questionnaire is a reliable and valid indicator of vision-related quality of life in patients with non-infectious uveitis and other ocular diseases [7-9].

The index analysis was performed to assess changes in QoL of patients receiving sirolimus as a therapy for non-infectious uveitis in the SAVE (Sirolimus as therapeutic Approach to uVEitis) study using patient-reported changes in QoL as an indicator.

## Methods

A randomized, open-label safety, and bioactivity clinical study was conducted at the Wilmer Eye Institute on 30 patients with non-infectious intermediate uveitis, posterior uveitis, and panuveitis in accordance with the SAVE Study protocol [10]. These patients were stratified at baseline on disease activity and the use of prednisone and/or other IMT agents into three categories: category 1: active uveitis and receiving no treatment; category 2: active uveitis and receiving ≥10 mg/day of prednisone and/or at least one other systemic immunosuppressant; or category 3: inactive uveitis and receiving <10 mg/day of prednisone and/or at least one other systemic immunosuppressant. Patients were required to discontinue all systemic immunosuppressants other than corticosteroids 30 days prior to the first study drug administration at baseline.

Active disease was defined as having at least 1+ vitreous haze using the Standardized Uveitis Nomenclature (SUN) Working Group scale and/or at least 1+ vitreous cell count using the Foster and Vitale scale. Inactive disease was defined as having vitreous haze of 0.5+ or less and vitreous cell count of 0.5+ or less, using the SUN Working Group and Foster and Vitale scales.

Patients were randomized and divided into two treatment groups. Group 1 received intravitreal (IVT) injections of 352 μg of sirolimus in the study eye on days 0, 60, and 120. Group 2 received subconjunctival (SCJ) injections of 1,320 μg of sirolimus in the study eye on days 0, 60, and 120. Starting at day 180, study subjects were eligible to receive additional subconjunctival or intravitreal sirolimus, based on their initial group randomization, every 2 months if they were found to have active disease as defined above. The end-of-study visit was at month 12.

The NEI VFQ 39 was self-administered at baseline (BL), month 6 (M6), and month 12 (M12) of the study. Patient composite and subscale scores were calculated according to the protocol in the NEI VFQ Manual [11]. The mean scores of all subscales were calculated for each category. All items are scored so that a high score represents better functioning, for example, a high ocular pain score would indicate minimal pain experienced by the patient. Each item is then converted to a 0 to 100 scale so that the lowest and highest possible scores are set at 0 and 100 points, respectively. The composite score was calculated by averaging the scores of the 12 subscales.

Pooled patient data was analyzed to assess response to study treatment regardless of study group or category. Subsequently, patient data was divided into treatment groups (SCJ vs IVT) and treatment categories and then analyzed for differences in subscale and composite scores.

The Wilcoxon signed-rank test was used to determine significance of changes between baseline, month 6, and month 12 for all groups. A 95% confidence interval with a 5% level of significance was used, therefore, a *P* value of <0.05 was considered significant.

## Results

The VFQ-39 was filled by 26 patients at BL and M6 and by 23 patients at M12. A total of six patients had exited the study before the M12 endpoint and one patient did not fill out the M12 questionnaire.

### Pooled data

Median subscale and composite scores for the pooled data are shown in Table 1. Significant improvements in scores were seen in the following subscales at both M6 and M12: ocular pain, distance activities, and vision-specific mental health. The pooled composite scores also showed significant improvements at both M6 and M12 (*P* < 0.001).

**Table 1:** **Pooled National Eye Institute 39-Question Visual Function Questionnaire composite and subscale scores**

	**Median**			***P*** **value**	
**VFQ-25 subscales**	**BL (** ***n*** **= 26)**	**M6 (** ***n*** **= 26)**	**M12 (** ***n*** **= 23)**	**BL-M6**	**BL-M12**
General health	69.6	71.7	71.6	0.51	0.28
General vision	62.3	70.4	71.7	*0.01*	0.07
Ocular pain	73.1	82.2	86.4	*0.03*	*<0.001*
Near activities	70.0	71.2	75.0	0.65	0.12
Distance activities	73.8	79.7	81.3	*0.03*	*0.02*
Vision-specific social functioning	87.5	91.3	94.0	0.21	*0.02*
Vision-specific mental health	59.5	69.4	76.3	*<0.001*	*<0.001*
Vision-specific role difficulties	70.7	76.2	82.9	0.07	*0.02*
Vision-specific dependency	82.9	88.0	92.7	0.09	*0.03*
Driving^a^	69.0	72.1	75.0	0.31	0.10
Color vision	90.4	95.2	94.6	0.23	0.16
Peripheral vision	78.8	82.7	79.3	0.39	0.75
Composite score for VFQ-39 (VFQ-25 + additional items)	74.3	79.9	82.8	*<0.001*	*<0.001*

Italicized values indicate statistical significance.

^a^Driving: *n* = 21 for BL and M6, *n* = 18 for M12.

BL, baseline; M6, month 6; M12, month 12, VFQ, Visual Function Questionnaire.

### Treatment groups

From BL to M12, patients in both groups reported a significant increase (improvement) in the ocular pain scores. However, group 1 also reported significant improvements in the areas of general vision, near activities, distance activities, vision-specific social functioning, and vision-specific mental health. Only group 1 displayed significant improvement in composite score from BL to M12 (*P* = 0.01) (Table 2).

**Table 2:** **The National Eye Institute 39-Question Visual Function Questionnaire composite and subscale scores divided by treatment group**

	**Group 1**					**Group 2**				
	**Median**			***P*** **value**		**Median**			***P*** **value**	
**VFQ-25 Subscales**	**BL** **(** ***n*** **= 13)**	**M6 ** **(** ***n*** **= 13)**	**M12** **(** ***n*** **= 12)**	**BL-M6**	**BL-M12**	**BL** **(** ***n*** **= 13)**	**M6 ** **(** ***n*** **= 13)**	**M12** **(** ***n*** **= 11)**	**BL-M6**	**BL-M12**
General health	73.08	73.08	71.43	1.00	0.80	66.21	70.33	71.75	0.47	0.30
General vision	59.49	70.77	72.78	*0.03*	*0.04*	65.13	70.00	70.61	0.18	0.70
Ocular pain	78.85	79.81	88.54	0.79	*0.01*	67.31	84.62	84.09	*0.01*	*0.01*
Near activities	66.35	67.69	76.18	0.73	*0.03*	73.59	74.74	73.64	0.78	0.56
Distance activities	73.33	79.17	83.96	0.15	*0.04*	74.36	80.29	78.41	0.12	0.23
Vision-specific social functioning	85.26	89.10	95.49	0.44	*0.03*	89.74	93.59	92.42	0.31	0.28
Vision-specific mental health	59.68	68.08	78.33	0.07	*0.01*	59.23	70.77	74.09	*0.02*	0.06
Vision-specific role difficulties	71.63	75.48	85.42	0.42	*0.04*	69.71	76.92	80.11	0.07	0.22
Vision-specific dependency	80.29	87.02	92.71	0.16	0.07	85.58	88.94	92.61	0.38	0.26
Driving^a^	79.63	81.48	80.21	0.56	0.52	64.58	72.22	70.00	0.08	0.54
Color vision	88.46	94.23	95.83	0.39	0.10	92.31	96.15	93.18	0.44	1.00
Peripheral vision	78.85	81.73	83.33	0.69	0.19	78.85	83.65	75.00	0.41	0.34
Composite score for VFQ-39 (VFQ-25 + additional items)	73.87	78.64	84.87	0.06	*0.01*	74.67	81.17	80.49	*0.03*	0.10

Italicized values indicate statistical significance.

^a^Driving (group 1): *n* = 9 for BL-M6 and *n* = 8 for BL-M12 comparison. Driving (group 2): *n* = 12 for BL-M6 and *n* = 10 for BL-M12 comparison.

BL, baseline; M6, month 6; M12, month 12, VFQ, Visual Function Questionnaire.

### Disease category

From BL to M12, patients in category 1 and 2 showed a significant increase (improvement) in ocular pain scores and vision-specific mental health scores. However, only patients in category 1 showed a significant increase in the NEI VFQ-39 composite scores at month 12 (*P* = 0.03) (Table 3).

**Table 3:** **Th** **e National eye institute 39-question visual function questionnaire scores analyzed by disease category**

	**Category 1**					**Category 2**					**Category 3**				
	**Mean**			***P*** **value**		**Mean**			***P*** **value**		**Mean**			***P*** **value**	
**VFQ-25 subscales**	**BL ** **(** ***n*** **= 7)**	**M6 ** **(** ***n*** **= 7)**	**M12** **(** ***n*** **= 4)**	**BL-M6**	**BL-M12**	**BL ** **(** ***n*** **= 11)**	**M6 ** **(** ***n*** **= 11)**	**M12 ** **(** ***n*** **= 11)**	**BL-M6**	**BL-M12**	**BL ** **(** ***n*** **= 8)**	**M6** ** (** ***n*** **= 8)**	**M12** **(** ***n*** **= 8)**	**BL-M6**	**BL-M12**
General health	73.98	73.47	73.21	0.93	0.39	76.62	75.32	74.03	0.73	0.44	56.25	65.18	67.41	0.26	0.11
General vision	50.48	58.57	70.00	0.38	0.08	66.06	76.36	73.33	*0.03*	0.27	67.50	72.50	70.42	0.14	0.65
Ocular pain	64.29	78.57	78.13	0.10	*0.04*	80.68	87.50	94.32	0.14	*0.001*	70.31	78.13	79.69	0.43	0.27
Near activities	54.52	57.98	63.33	0.66	0.12	73.48	76.89	79.17	0.23	0.16	78.65	75.00	75.00	0.49	0.30
Distance activities	64.76	69.35	71.88	0.52	0.06	77.27	84.09	82.58	*0.01*	0.29	77.08	82.81	84.27	0.36	0.18
Vision-specific social functioning	79.76	84.52	83.33	0.52	0.10	87.12	94.70	96.21	0.10	0.07	94.79	92.71	96.35	0.68	0.55
Vision-specific mental health	34.76	47.14	51.25	0.16	*0.05*	64.09	73.18	81.36	0.07	*0.02*	74.69	83.75	81.88	*0.02*	0.30
Vision-specific role difficulties	47.32	52.68	64.06	0.36	0.11	80.11	85.80	89.20	0.22	0.19	78.13	83.59	83.59	0.41	0.33
Vision-specific dependency	66.96	74.11	75.00	0.31	0.10	82.39	88.64	94.89	0.27	0.13	97.66	99.22	98.44	0.45	0.60
Driving	56.55	55.56	47.22	1.00	0.42	81.48	74.24	78.03	0.36	0.49	65.48	84.72	83.33	*0.03*	0.08
Color vision	85.71	92.86	93.75	0.46	0.18	88.64	95.45	93.18	0.39	0.44	96.88	96.88	96.88	NS	NS
Peripheral vision	75.00	82.14	75.00	0.57	0.64	84.09	87.50	84.09	0.57	1.00	75.00	76.56	75.00	0.83	1.00
Composite score for VFQ-39 (VFQ-25 + additional items)	61.83	68.70	71.09	0.21	*0.03*	78.18	84.03	86.03	*0.03*	0.08	79.78	84.04	84.13	0.15	0.09

Italicized values indicate statistical significance.

BL, baseline; M6, month 6; M12, month 12, VFQ, Visual Function Questionnaire, Category 1, active uveitis and receiving no treatment; Category 2, active uveitis and receiving ≥10 mg/day of prednisone and/or at least one other systemic immunosuppressant; Category 3, inactive uveitis and receiving <10 mg/day of prednisone and/or at least one other systemic immunosuppressant.

## Discussion

In this QoL analysis of study subjects in the SAVE Study, NEI VFQ-39 scores have demonstrated that both intravitreal and subconjunctival injections of sirolimus were well tolerated. After 12 months, pooled data from patients treated with sirolimus (regardless of the mode of injection) showed a statistically significant improvement in composite scores for NEI VFQ-39 (*P* < 0.001). Specifically, patients showed greatest score improvements in the subscales of ocular pain and vision-specific mental health (*P* < 0.001). Patients in group 2 showed a greater increase in ocular pain scores from BL to month 6; however, the effect seemed to have plateaued off and no significant improvement was observed in this group beyond month 6. Group 1 continued to show improvement until month 12. Improvements were also reported in vision-specific social functioning, vision-specific role difficulties, and vision-specific dependency.

When divided into treatment groups, it is apparent that IVT injection of sirolimus showed a significant improvement in a higher number of subscales at M12 compared to SCJ administration. Patients receiving sirolimus injections in both the groups showed a significant improvement in ocular pain after 12 months (*P* < 0.01). In addition, patients receiving IVT injections (Group 1) show significant improvements in overall vision-related functioning after 12 months compared to baseline. Results indicate that sirolimus treatment provides a significant decrease in ocular pain regardless of route of administration. However, IVT injection results in improvement in the additional areas of general vision, near activities, distance activities, vision-specific social functioning, and vision-specific role difficulties.

In patients that received sirolimus subconjunctivally, the most frequently reported adverse event was inflammation at the injection site, manifesting as ocular discomfort; conjunctival hyperemia and chemosis [6]. Such adverse events are possible factors as to why patient-reported QoL scores from patients receiving subconjunctival injections would be lower than those receiving intravitreal injections.

Analyzing results by disease category showed that patients in Category 1 exhibited the greatest increase in Qol scores after treatment with sirolimus. Patients in this category had active uveitis and were not receiving any treatment. It is possible that the absence of treatment in these patients may have caused the substantially lower VFQ scores at BL and therefore possibly providing more room for improvement with sirolimus treatment. Conversely, improvements in QoL scores may have been masked in patients that were using corticosteroids (categories 2 and 3) due to steroid-related adverse events over the course of the study.

While sirolimus has demonstrated bioactivity as an IMT and corticosteroid-sparing agent to treat non-infectious uveitis, the analyses from our NEI VFQ-39 assessments have indicated that patients receiving sirolimus also show an overall improvement of vision-related health.

Our study is among the very first being reported in the literature on the assessment of QoL in patients with uveitis undergoing therapy with local administration of an IMT. An important limitation of our study is the small sample size. Due to the small sample size (*n* = 26), it is not certain that these results are applicable to the larger population of patients with non-infectious uveitis. Currently, randomized phase 2 and phase 3 studies of intravitreal sirolimus in non-infectious uveitis are being conducted in the United States and throughout the world to investigate the efficacy of sirolimus. Additional QoL analyses of patients enrolled in these larger randomized control trials with sirolimus will be very helpful to determine if sirolimus is effective in not only controlling the disease but also beneficial in improving the quality of life of patients suffering from non-infectious uveitis.

## Conclusions

Locally delivered sirolimus has demonstrated bioactivity as an IMT and corticosteroid-sparing agent to treat non-infectious uveitis. Patients receiving intravitreal injection of sirolimus showed overall improvement of vision-related health while those receiving subconjunctival injections did not. Larger randomized control trials with sirolimus are indicated to validate these results.

## Abbreviations

BL: baseline
IMT: immunomodulatory therapeutic
IVT: intravitreal
M6: month 6
M12: month 12
NEI VFQ-39: National Eye Institute Visual Function Questionnaire 39-Item
QoL: quality of life
SAVE: Sirolimus as therapeutic Approach to uVEitis
SCJ: subconjunctival
SUN: Standardized Uveitis Nomenclature
